# Systematical Engineering of Synthetic Yeast for Enhanced Production of Lycopene

**DOI:** 10.3390/bioengineering8010014

**Published:** 2021-01-19

**Authors:** Yu Zhang, Tsan-Yu Chiu, Jin-Tao Zhang, Shu-Jie Wang, Shu-Wen Wang, Long-Ying Liu, Zhi Ping, Yong Wang, Ao Chen, Wen-Wei Zhang, Tai Chen, Yun Wang, Yue Shen

**Affiliations:** 1 BGI Education Center, University of Chinese Academy of Sciences, Shenzhen 518083, China; zhangyu6@genomics.cn; 2 BGI-Shenzhen, Beishan Industrial Zone, Shenzhen 518083, China; qiucanyu@genomics.cn (T.-Y.C.); wangshujie1@genomics.cn (S.-J.W.); wangshuwen0202@163.com (S.-W.W.); liulongying@jcmsc.cn (L.-Y.L.); pingzhi@genomics.cn (Z.P.); wangyong4@genomics.cn (Y.W.); chenao@genomics.cn (A.C.); zhangww@genomics.cn (W.-W.Z.); wangyun@genomics.cn (Y.W.); 3 Guangdong Provincial Key Laboratory of Genome Read and Write, BGI-Shenzhen, Shenzhen 518120, China; zhangjintao@cngb.org (J.-T.Z.); chentai@cngb.org (T.C.); 4 Guangdong Provincial Academician Workstation of BGI Synthetic Genomics, BGI-Shenzhen, Shenzhen 518120, China; 5 China National GeneBank, BGI-Shenzhen, Jinsha Road, Shenzhen 518120, China; 6 Shenzhen Institute of Synthetic Biology, Shenzhen Institutes of Advanced Technology, Chinese Academy of Sciences, Shenzhen 518000, China

**Keywords:** synthetic yeast, SCRaMbLE, combinatorial assembly, lycopene production optimization

## Abstract

Synthetic biology allows the re-engineering of biological systems and promotes the development of bioengineering to a whole new level, showing great potential in biomanufacturing. Here, in order to make the heterologous lycopene biosynthesis pathway compatible with the host strain YSy 200, we evolved YSy200 using a unique Synthetic Chromosome Rearrangement and Modification by LoxP-mediated Evolution (SCRaMbLE) system that is built in the Sc2.0 synthetic yeast. By inducing SCRaMbLE, we successfully identified a host strain YSy201 that can be served as a suitable host to maintain the heterologous lycopene biosynthesis pathway. Then, we optimized the lycopene biosynthesis pathway and further integrated into the rDNA arrays of YSy201 to increase its copy number. In combination with culturing condition optimization, we successfully screened out the final yeast strain YSy222, which showed a 129.5-fold increase of lycopene yield in comparison with its parental strain. Our work shows that, the strategy of combining the engineering efforts on both the lycopene biosynthesis pathway and the host strain can improve the compatibility between the heterologous pathway and the host strain, which can further effectively increase the yield of the target product.

## 1. Introduction

As a plant derived nutrient with antioxidant properties, lycopene is commonly used as a food additive [1] and its biosynthesis pathway has been well-characterized [2]. Production of lycopene using microbial sources, such as *Blakeslea trispora* [3,4], *Escherichia coli* [5,6,7,8,9], and *Saccharomyces cerevisiae* [10,11,12,13,14], is presently of great interest. By random mutagenesis against *B. trispora* on its whole genome and fermentation optimization, the yield of lycopene has been increased up to 944.8 mg/L [3]. Since *E. coli* cannot produce lycopene naturally, extensive metabolic engineering efforts, such as whole pathway engineering, cofactor engineering, membrane engineering, and directed evolution have been conducted to reach the lycopene yield of 448 mg/g dry cell weight (DCW) [5,6,7,8,9]. However, microbial production of lycopene in *B. trispora* [15] or *E. coli* has its own shortcomings due to food safety issues [16]. Thus, engineering yeast, especially *S. cerevisiae* for increased lycopene production, have also been investigated extensively [10,11,12,13,14].

*S. cerevisiae*, as a microbial factory, the heterologous lycopene biosynthetic pathway needs to be expressed and coordinated with its native metabolism. Lycopene is a type of tetraterpenoid (C40) consisting of the core C5 isoprene units derived from the native mevalonate (MVA) pathway [17] (Figure S1). The endogenous pathway ends with geranylgeranyl diphosphate (GGPP) as the initial precursor of terpenoids. Further, the expression of the exogenous genes *CrtE* (GGPP synthase), *CrtB* (phytoene synthase), and *CrtI* (phytoene desaturase) converts GGPP to lycopene as the end product. In order to optimize the heterologous lycopene biosynthetic pathway, pathway engineering has been done, in regards to increasing the production of a key precursor by overexpressing limiting enzymes in the pathway, increasing NADPH generation synthesis to fuel the production pathway, and bioprospecting lycopene biosynthetic genes from various origins to improve catalytic activities [10,11,12,13,14]. In addition to pathway engineering, increasing cell fitness or stress tolerance can also contribute to improve product yields. The toxicity of lipophilic lycopene is known to cause membrane stresses in non-oleaginous *S. cerevisiae*, which can be relieved by supplementing linoleic acid in the media, by engineering fatty acid associated pathway or inducing product production at the desire conditions with an inducible promoter [10,11]. With these rational designed approaches, the highest lycopene yield in *S. cerevisiae* achieved up to date is 3.28 g/L in a 7 L fermenter [10]. It is worth noting that the desired strain for practical industrial application requires efforts in the pathway optimization and host strain engineering. In the recent study, even with comprehensive optimization against a lycopene biosynthesis pathway and the *S. cerevisiae* host strain, the significant increment of lycopene yield is still largely dependent on the employment of the *GAL*-regulation system, which let the high cell density be achieved first, and sequentially led to high lycopene production [10]. This indicates that the potential of the host strain is still under-explored. Predictably, it would be even more challenging if little is known of the functional related metabolism network of host strain.

The development of DNA synthesis and genome editing tools has rapidly reformed the synthetic genomics field. Sc2.0 is an international milestone project that aims to redesign and rebuild yeast chromosome with better stability and flexibility without drastically changing the cell fitness. In the past few years, Sc2.0 made great progress in creating designer yeasts harboring one, or multiple, synthetic chromosomes [18,19,20,21,22,23,24]. The Synthetic Chromosome Rearrangement and Modification by LoxP-mediated Evolution (SCRaMbLE) system is a unique built-in tool, in which the 34 bp loxPsym recombination site is strategically placed at downstream of all nonessential genes in the redesigned chromosomes, to increase the genomic content diversity [25]. Upon estradiol induction, the cyclization recombination enzyme (Cre) fused to an estradiol-binding domain (EBD) will be translocated into the nucleus and recognize loxPsym sites to facilitate combinatorial chromosomal rearrangements, such as insertion, deletion, duplication, or translocation between any two loxPsym sites, and generate strain libraries with phenotypes that differ from their parent under different conditions [25]. SCRaMbLE has been applied in yeast strains engineering for various purposes, such as to improve ethanol tolerance [26], or to increase the productivity of beta-carotene [27], violacein [28], and other chemicals of industrial interest [29]. This approach not only speeds up the process of optimal yeast strain screening, but also helps researchers gain new insights into yeast mechanisms through identifying the important gene(s) in chromosomal rearrangements that play a major role in the improved yeast phenotype.

Here, in our study, we explored the potential of utilizing this unique system built in the synthetic yeast strain to quickly improve lycopene production in *S. cerevisiae.* By doing so, we successfully screened out a SCRaMbLEd host strain with optimized lycopene biosynthesis pathway and proved that the lycopene yield can be effectively increased by 129.5-fold, in comparison with its parental strain. Genomic and transcriptome analyses have helped us to understand the potential mechanism on how the observed chromosomal combinatorial rearrangements facilitate the increase of lycopene production.

## 2. Materials and Methods

### 2.1. Strains and Media

The yeast strains used in this study are listed in Supplementary Table S1. The previously constructed yeast strain synII (*MAT***a** *his3Δ1 leu2Δ0 ura3Δ0 met15Δ0*), in which chromosome II being replaced by its synthetic version chromosome, is used in this study [18]. Yeasts were cultured in Yeast Extract Peptone Dextrose (YPD) medium (20 g/L peptone, 10 g/L yeast extract, and 20 g/L glucose) or SC medium (synthetic complete medium lacking appropriate amino acids with 20 g/L glucose), or 500 mg/L G418 Sulfate (GENVIEW) was added in media for KanMX marker screening.

*Escherichia coli* DH5α strains, which were used for plasmid construction, were cultured in Luria-Bertani (LB) media (10 g/L peptone, 5 g/L NaCl, 5 g/L yeast extract) with 50 µg/mL of ampicillin or 50 µg/mL of kanamycin supplemented for selection.

### 2.2. Plasmids and Strains Construction

The plasmids and primers used in this study are listed in Supplementary Tables S2 and S3, respectively. DNA polymerase, restriction endonucleases, and T4 ligase were purchased from New England Biolabs. The DNA sequences of four lycopene synthetic pathway genes, including geranylgeranyl diphosphate synthase (CrtE) from *Xanthophyllomyces dendrorhous*, 15-cis-phytoene synthase (CrtB) from *Pantoea ananatis*, phytoene desaturase (CrtI) from *Xanthophyllomyces dendrorhous*, and isopentenyl-diphosphate delta-isomerase (IDI) from *E. coli*, were codon-optimized and synthesized, and then ligated into HcKan_O vector by YeastFab method [30]. Via this method, *TEF2p* promoter and *ADH1t* terminator were amplified from BY4741 genomic DNA, and then ligated into HcKan_P or HcKan_T vector. The promoter and terminator were assembled with each gene to four lycopene pathway transcriptional units on Promoter-Open reading frame-Terminator (POT) receiving vector POT, and then assembled into vector pRS416 to generate the plasmid pYS001.

To regulate and optimize the pathway genes expression level, we collected a series of different strength promoters (*CYC1p*, *TEF2p*, *TDH3p*, *CCW12p*, *PGK1p*, *RPL18bp*), and four sequence-distinct terminators (*CIT1t*, *FUM1t*, *ERP2t*, and *ADH1t*) from yeast. Then, the four lycopene biosynthesis genes were assembled, with one of each promoter and terminator from the collection to form transcriptional units in the POT vector. Next, the POT plasmids carrying various transcriptional units were mixed together for further pathway assembly through the YeastFab method [30]. Screening was performed against the gradation of pigment.

For the deletion of one copy *PEX32* gene in the SCRaMbLEd strain YSy200, the *LEU2* marker gene was used to replace the entire coding region of *PEX32* gene. The 480 bp upstream and 296 bp downstream homologous sequence in the untranslated region of *PEX32* gene were amplified from the YSy200 genomic DNA, then transferred together with *LEU2* fragment into YSy200 strain. PCR primers that can bind specifically to PEX32 and LEU2 coding regions were designed, respectively, for validation.

The 500 bp upstream sequence of rDNA25 (rDNA25L), *KanMX* marker, *CCW12p* promoter, the 500 bp downstream sequence of rDNA25 (rDNA25R), *ADH1t* terminator were amplified from yeast genome or synthesized, and then assembled by overlap extension PCR (OE-PCR), obtaining the upstream genomic integrated fragment rDNA25L-KanMX-*CCW12p* and the downstream genomic integrated fragment *ADH1t*-rDNA25R, respectively. These integrated fragments and enzyme digestion products of the best lycopene plasmid were integrated into yeast genomic rDNA25 multiple copy sites via homologous recombination of *Saccharomyces cerevisiae* in vivo [31].

All yeast transformations were performed using the lithium acetate method [32]. To construct diploid strain, synII (*MAT***a**) and BY4742 (*MAT*α) single colonies were picked and inoculated into YPD medium overnight at 30 °C and 220 rpm, respectively. The precultures were inoculated into new YPD medium together with initial OD_600_ = 0.1 overnight at 30 °C and 220 rpm. Then the cultures were spread on SC-Met-Lys selective plate followed by 3 days incubation at 30 °C and verified by PCR with mating type verification primers binding to *MATα*/*MAT***a** loci.

### 2.3. Lycopene High-Yield Strains SCRaMbLE and Screening

Before SCRaMbLE, the pRS413-CreEBD plasmid [33] was transformed into lycopene-producing strain YSy200 and selected on SC-His-Ura selective plate. The single colony was inoculated into 3 mL SC-His-Ura liquid medium overnight at 30 °C, 220 rpm. The precultures were transferred into flasks containing 50 mL of fresh medium with an initial OD_600_ = 0.1. The 17β-Estradiol (Toronto Research Chemicals, North York, ON, Canada) was added to a final concentration of 1 µM and cultures were grown shaking at 30 °C, 220 rpm for 24 h to induce SCRaMbLE. After 24 h induction, 1 mL of culture was plated on SC-Ura agars and incubated at 30 °C for 3 days. The colonies display darker color were selected for further characterization.

### 2.4. Lycopene Extraction and Quantification

A single colony of the candidate strains to be fermented were inoculated into 3 mL SC-Ura medium at 30 °C, 220 rpm for 24 h, and then the preculture was transferred into a 14 mL shaking tube containing 5 mL fresh SC-Ura with an initial OD_600_ = 0.1. The batch cultivation was conducted at 30 or 20 °C, 220 rpm for 5 days.

After fermentation, 1 mL of cell culture was collected by centrifugation at 8000× *g* rpm for 5 min and washed with deionized water. The cells were resuspended with 0.5 mL acetone and petroleum ether (9:1) mixture and broken with glass beads (1 g, 0.50–0.75 mm) by vortex for 10 min. The mixture was centrifuged at 12,000× *g* rpm for 5 min and the supernatant was transferred to a new tube. The extraction was repeated by adding 0.5 mL acetone and petroleum ether (9:1) mixture again and sonicated until the pellets were colorless.

Lycopene was analyzed using reversed-phase high performance liquid chromatography using isocratic elution and UV detection at 472 nm (Agilent 1260 Infinity II). A YMC Carotenoid column (250 mm × 4.6 mm, S-5 µm, Catalog number: CT99S05-2546WT) was used. Mobile phases A (methanol/MTBE/water = 81/15/4, *v*/*v*) and B (methanol/MTBE/water = 6/90/4, *v*/*v*) were eluted as 5%, 100%, and 100%, at 0, 7 and 15 min (MTBE: methyl tert-butyl ether). The flow rate was 1 mL/min column temperature was 30 °C and the injection volume 30 µL.

### 2.5. Genomic DNA Extraction

Yeast cells were inoculated in 20 mL SC-Ura medium using a 50 mL tube for 2 days. Pellets were collected by centrifugation at 8000× *g* rpm for 2 min then washed once using 1 M sorbitol. Moreover, 600 μL 1 M sorbitol was added to resuspend the pellet and 750 U lyticase (Sigma) was added. Finally, shock treatment was performed at 37 °C 800 rpm for more than 1 h. Pellets were collected by centrifugation at 12,000× *g* rpm for 2 min and added 600 μL Triton-SDS lysis buffer was treated at 65 °C 600 rpm for 30 min. 600 μL PCI (Phenol:Chloroform:Isoamyl alcohol = 25/24/1, *v*/*v*) was added, the mixture was mixed upside down, and then centrifuged at 12,000× *g* rpm for 20 min. 500 μL of the aqueous layer was transferred to a new 1.5 mL tube. The genomic DNA was precipitated by adding 500 μL of isopropyl alcohol and kept at room temperature for 5 min. Then genomic DNA was pelleted by centrifugation at 12,000× *g* rpm for 10 min. The precipitate was washed with 500 μL 75% ethanol, followed by 15 min drying at 37 °C. The genomic DNA was resuspended in 50 μL TER buffer (10 mM Tris-HCl pH8.0, 1 mM EDTA with 25 mg/mL RNase) and incubated at 37 °C for 2 h. 1 μL of product was analyzed on agarose gel electrophoresis.

### 2.6. Whole Genome Sequencing (WGS) and Data Processing

Library construction and whole genome sequencing were done with standard protocols with BGISEQ-500. Quality control of sequencing reads was performed before mapping. Reads with adapters or shorter than 100 bp were removed. Reads containing more than 1% of unknown base or containing more than one base with Phred-score lower than 10 were removed. After barcode deconvolution, 1.6 Gbp raw data per strain, representing 78-fold clean coverage of the yeast genome, was obtained. Filtered reads were mapped to the synII reference sequences using the short-read aligner of Short Oligonucleotide Alignment Program (SOAP) (version 2.21) [34]. Splitting reads for junction identification and detection of structural variation were performed according to the method [25].

### 2.7. RNA Extraction and Transcriptome Analysis

To prepare lycopene high-yield strains RNA, we used the Yeast RNA kit (OMEGA BioTek) according to the manufacturer’s instructions. Raw sequencing reads from DIPSEQ-T1 were filtered using SOAPnuke (version 2.1) [35]. Reads left after filtration were mapped to the genome (BY4741 and synII) using hisat2 (version 2.1.0) [36] (run with the -k 1 parameter) and sorted by samtools (version 1.7) [37]. Transcripts per Kilobase Million (TPM) value was used to estimate the gene expression abundance, which was calculated by StringTie (version 2.1.2) [37] (run with the -e -B parameter).

### 2.8. Gene Differential Expression and Pathway Enrichment Analysis

The fold change for ranking differentially expressed genes (GFOLD) (version 1.1.4) [37] was used to assign reliable statistics for expression changes derived from RNA-seq data based on the posterior distribution of log fold change. The GFOLD value (log2 fold change) of 1 was set as threshold for scoring of differential expression. The genes with absolute value of log2 (fold-change) greater than 0 were subjected to functional enrichment analysis, in which log2 (fold-change) greater than 0 was considered upregulated and log2 (fold-change) less than 0 was considered downregulated. The functional enrichment was performed in 3 categories of Gene Ontology (GO) terms: biological process (BP), molecular function (MF), and cellular component (CC). Kyoto Encyclopedia of Genes and Genomes (KEGG) pathway enrichment was also performed. The *p* value was calculated using hypergeometric test. The *p* value < 0.05 was considered to indicate a statistically significant difference.

### 2.9. Reverse Transcription and Quantitative PCR

To quantify the expression level of *CrtE*, *CrtB*, *CrtI*, and *IDI* genes in the constructed plasmid (pYS010), the total RNA was extracted from strain YSy212 and using BY4741 as control. The cDNA was synthesized with PrimeScript™ RT reagent kit with gDNA Eraser (Takara, cat #RR047A) following the standard protocol. The *ACT1* was used as reference gene [38]. The assay was performed on StepOnePlus Real-Time PCR System with TB Green^®^ Premix Ex Taq™ II (Tli RNase H Plus) (Takara, cat# RR820A). The relative expressions were quantified by comparing the C_T_ values of the target genes and the reference gene using the 2^−ΔΔCt^ method [39]. The primers for qPCR are listed in Supplementary Table S3.

### 2.10. Growth Assay

A single colony of candidate strains was isolated from agar plate and inoculated into 3 mL SC-Ura medium at 30 °C, 220 rpm for 24 h, and then subcultured into a Honeycomb plate containing 200 μL fresh SC-Ura liquid medium with an initial OD_600_ = 0.01. Then continuous measurement of OD_600_ by Bioscreen C MBR (Oy Growth Curves Ab Ltd., Finland) at 30 °C was performed for 2 days.

### 2.11. Pathway Stability Assay

Single colonies of the candidate strains were inoculated in 3 mL SC-Ura medium at 30 °C, 220 rpm for 24 h, and then subcultured in a 14 mL shaking tube containing 5 mL fresh SC-Ura liquid media with the initial OD_600_ at 0.1. The culture was incubated at 30 °C, 220 rpm for 24 h, and then plated on SC-Ura agar plates and incubated at 30 °C for 3 days. All red and white colonies in each plate were counted respectively to analyze the stability of constructed pathway: the proportion of red colonies (%) = total number of red colonies/(total number of white colonies + total number of red colonies).

### 2.12. Copy Number Estimation of Integrated Pathway by Quantitative PCR

To assay copy numbers of integrated pathway in the rDNA array, yeast genomic DNA was used for qPCR analysis. The *RFA1* gene was chosen as the reference gene and the copy number of the pathway was measured following the method described in previous research [40]. The copy numbers of strain YSy221 and YSy222 are 3.91 ± 2.32 and 4.26 ± 2.67, respectively (n = 3).

## 3. Results

### 3.1. SCRaMbLE to Generate a Yeast Strain with Increased Lycopene Production

**Figure 1:**
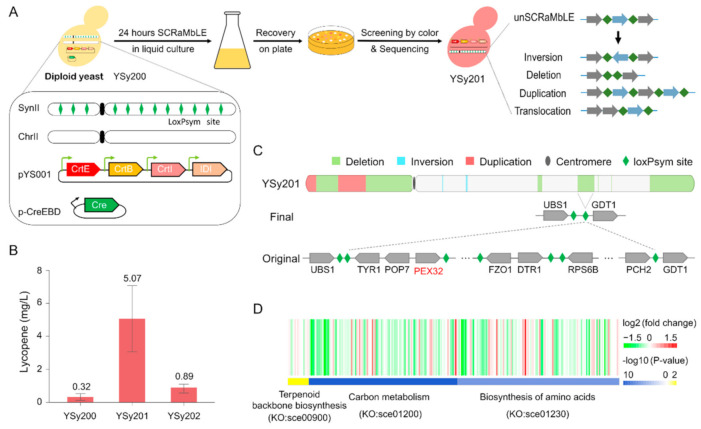
Host strain improvement by Synthetic Chromosome Rearrangement and Modification by LoxP-mediated Evolution (SCRaMbLE) of diploid yeast bearing synthetic chromosome. (**A**). Schematic illustration of host optimization by SCRaMbLE for improving lycopene production in *S. cerevisiae*. (**B**). The lycopene production in strain YSy200 (unSCRaMbLEd), YSy201(SCRaMbLEd) and YSy202 (YSy200 pex32*Δ0*/PEX32) were assessed by HPLC. Error bars indicate ± SD (n = 3). (**C**). The structure variations created by SCRaMbLE in strain YSy201. (**D**). Three key pathways (KO:sce00900, KO:sce01200 and KO:sce01230) display transcriptional changes in strain YSy201.

For SCRaMbLE of host strain, loxPsym mediated recombination may create various genetic diversities, such as deletion, inversion, and duplications (Figure 1A). Even the loxPsym sites are designed to locate at the downstream of non-essential genes, the recombination between loxPsym sites may lead to loss of large fragments, which contain essential genes. This would drastically reduce the viability of a haploid yeast and potentially decrease the diversity generated by SCRaMbLE, while the random nature of SCRaMbLE events can be reserved in heterozygous diploid [41]. In addition, heterosis is known to improve function of any biological quality in a hybrid offspring due to mixing the genetic contributions of the pair of the chromosomes [42]. Thus, in our study, we initiate the host strain optimization by SCRaMbLEing in a diploid strain YSy200 generated by mating previously constructed haploid Sc2.0 yeast strain bearing the synthetic chromosome II to native yeast strain BY4742. The pYS001 plasmid containing lycopene biosynthesis pathway driven by *TEF2p* promoter was transformed into the diploid strain, with the initial lycopene yield of 0.32 mg/L (Figure 1B). By screening against pigment density of colonies recovered after SCRaMbLE, we identified a yeast strain YSy201 with 15.8-fold (*t*-test one-sided *p*-value = 9.1 × 10^−3^) increase of lycopene yield of 5.07 mg/L (Figure 1B).

Whole genome sequencing of YSy201 revealed varying structure rearrangements evenly distributed across the entire synthetic chromosome II, including two duplications, two inversions, and seven deletions (Figure 1C, Supplementary Table S4). We systematically checked the genes among all structural variation areas, and in one deletion area, we found one potential target gene. *PEX32*, which might play an essential role in improving the lycopene yield (Figure 1C). The *PEX32* gene is known to play roles in peroxisome proliferations and previous study has been demonstrated that disruption of *PEX30-32* increases peroxisome numbers and size, which may benefit fatty acid derived products [43]. In addition, Zhou et al. increased the number of peroxisomes per cell, having, as a result, the improvement of alkane titers up to three-fold by deletion of *PEX31* and *PEX32* in addition to overexpression of *PEX34* [44]. This leads to the question that whether the observed increasement of lycopene yield is mainly dependent on the deletion of *PEX32* gene in SCRaMbLEd strain YSy201. In order to verify this hypothesis, we knockout exactly one copy of *PEX32* gene in the unSCRaMbLE diploid strain YSy200. The resulting strain YSy202 with one copy of *PEX32* gene shows a 2.8-fold increase of lycopene yield, in comparison with its parental strain (Figure 1B). Our result suggests that the functional disruption of *PEX32* gene seems to be a contributing, but not exclusive, factor to the increase of lycopene yield.

To further elucidate the impact of chromosomal rearrangement events to the transcriptional changes, which leads to lycopene yield improvement, we performed transcriptome profiling to YSy201 in comparison with its parental strain YSy200. A total of 181 differentially expressed genes (DEGs, GFOLD value: log2 fold change > |1|) were identified (Supplementary Table S5). Results shows that the mevalonate pathway from Acetyl-CoA to Farnesyl diphosphate (FPP) in terpenoid backbone biosynthesis (KO:sce00900) is upregulated in YSy201 strain (*p*-value = 0.36, Figure 1D, Figure S1), suggesting that the more lycopene synthesis precursors are produced, the faster is the lycopene production. More experiments should have been done to confirm this hypothesis. In addition, we found that the carbon metabolism (KO:sce01200) and biosynthesis of amino acids (KO:sce01230) are significantly downregulated in YSy201, respectively with significance at a *p*-value of 1.14 × 10^−14^ and 1.13 × 10^−9^, comparing with its parental strain (Figure 1D). This might explain the slightly increase of doubling time by ~30% of YSy201 (Figure S2). A potential explanation for the observed biological process downregulation is that the metabolic flux distribution of the YSy201 strain is changed by a decreased level of a general cellular process, redirecting the flux towards the biosynthesis pathway of lycopene.

At this point, by combining genome and transcriptome analysis, we have shown that the new layout of SCRaMbLEd synthetic chromosome can lead to observed improvement for phenotypes of interest, which is the improvement of lycopene production. Moreover, the YSy201 can be used as a potential superior host strain for lycopene pathway integration.

### 3.2. Lycopene Biosynthesis Pathway Optimization by Combinatorial Assembly of Standard Biological Parts

The engineering of the target product biosynthesis pathway is not only for the purpose of yield improvement by fine-tuning the expression level of each gene in the pathway, but also for maintaining its stability in the host strain. It was previously reported that repetitive sequence is known to cause pathway instabilities in yeast due to high homologous recombination rates [45,46]. This phenomenon was further confirmed in our study when we performed host strain optimization by SCRaMbLE using the constructed pathway pSY001 containing the four genes, all driven by the same regulatory element *TEF2p* promoter. A significant portion of recovered colonies post-SCRaMbLE failed to produce lycopene, and further investigation suggests that the *CrtI* gene is frequently looped out due to the homologous recombination of the *TEF2p* promoter sequence between two transcriptional units, leaving the incomplete construct of lycopene biosynthesis pathway in the host strain (Figure S3).

**Figure 2:**
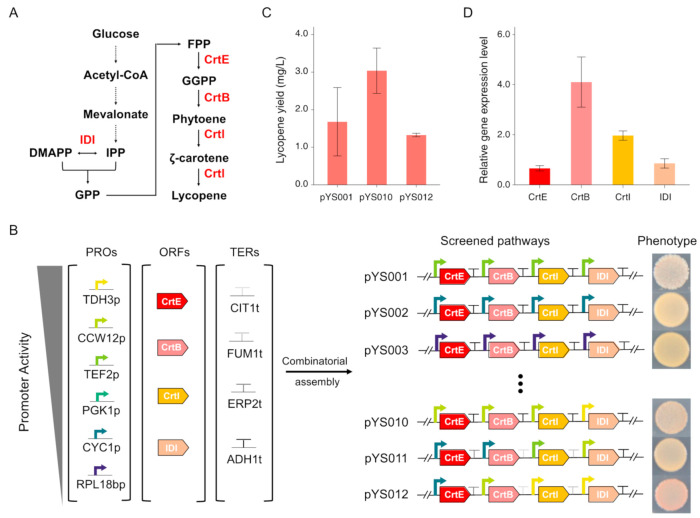
Lycopene biosynthesis pathway optimization by combinatorial assembly and screening. (**A**). Overview of lycopene biosynthesis pathway in *S. cerevisiae*. Key enzymes expressed for lycopene production are highlighted in red. (**B**). Schematic illustration of parts used for combinatorial assembly and assess their effects to lycopene production. (**C**). The lycopene yield of the strains hosting pYS001, pYS010, and pYS012 were measured by HPLC. (**D**). Evaluation of individual promoter strengths in strain YSy212 containing pYS010 plasmid by RT-PCR. Error bars indicate ± SD (n = 3).

The feasibility of combinatorial assembly of metabolic pathways, with modular designed biological parts, including promoters (PRO), open reading frames (ORF), and terminators (TER) has been demonstrated in *S. cerevisiae* [30]. Therefore, in our study, we aim to explore the potential of a similar approach for the purposes of optimal yield and stability in the host strain. Six well-characterized promoters with varying activities in *S. cerevisiae* [47,48] were selected for fine-tuning the expression level of the four transcriptional units to function coordinately for lycopene yield improvement. Four terminators in *S. cerevisiae* were also chosen to minimize the sequence similarity of each assembled transcriptional units to reduce the possibility of instability. Each biological part in our collection was designed with junction ends to adopt sequential assembly in the order of PRO-ORF-TER for each transcriptional unit and the distinct homology ends of each unit will further facilitate the full construction of lycopene biosynthesis pathway in BY4741 strain. By screening the constructions by the color, we observed a diverse combination of selected biological parts (Figure 2B). The exhibited shades of red pigment indicate the yield of lycopene in the observed colonies. After production quantification, we identified two constructs, pYS010 and pYS012, showing relatively higher lycopene yield of 3.04 and 1.33 mg/L, respectively (Figure 2C). Moreover, the lycopene production level of pYS010 seems more productive. RT-qPCR analysis towards the four target genes (Figure 2A) of pYS010 construct shows that in YSy212 strains, *CrtB* gene has the highest expression following the expression of *CrtE*, *CrtI*, and *IDI* (Figure 2D). To sum up, our result suggests that the combinatorial assembly strategy of the biosynthetic pathway can fine-tune the expression level of each gene in the pathway to improve the final yield.

### 3.3. Optimize Lycopene Production by Multicopy Integration of Constructed Pathway in SCRaMbLEd Host Strain

**Figure 3:**
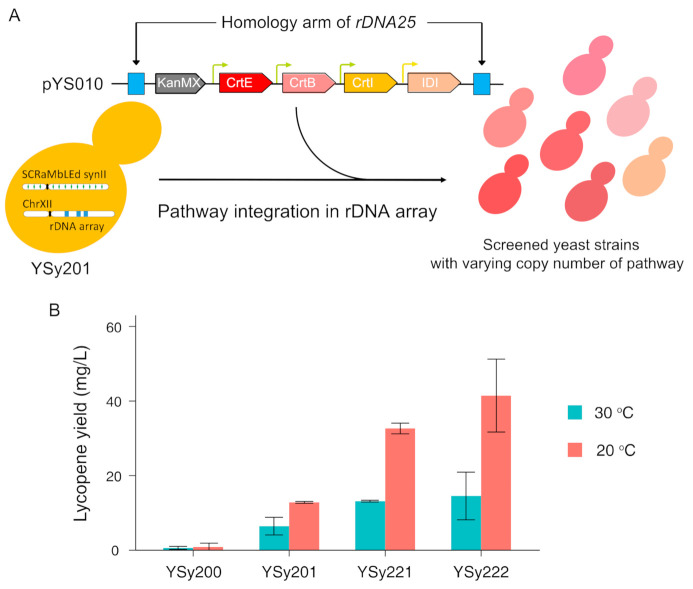
Characterization and improvement of lycopene -producing strain with optimized pathway. (**A**). Schematic illustration of host optimization by rDNA integration for improving lycopene production in *S. cerevisiae*. (**B**). Lycopene quantification of SCRaMbLEd strains and their variants, in comparison with the initial unSCRaMbLE strain YSy200. Error bars indicate ± SD (n = 3).

For the efficient production of target products, applying a high copy plasmid-based system is generally considered effective and convenient in expressing genes of interest involving the biosynthesis pathway. However, in industrial application, the use of plasmids has problems in unstable output of production caused by plasmid instability and extra manufacturing cost of selection markers during fermentation [49]. Chromosomal integration of heterologous constructs shows advantage in long-term stability and reduced cell-to-cell variability in copy number and expression levels. Therefore, this approach is more suitable for industrial-scale processes and has been widely adopted for downstream engineering in both bacterial and eukaryotic hosts [50]. In addition, the multicopy integration of heterologous genes into the ribosomal DNA for expression improvement has been reported in several yeasts [51,52]. With the constructed pathway pYS010, we tried to further increase its copy number by integrating into rDNA locus of generated SCRaMbLEd strain YSy201 (Figure 3A). The homology arms are designed at upstream and downstream of *rDNA25* to facilitate the integration of pYS010 construct. Through this approach, we identified two candidates with varying degrees of observable pigment density changes, indicating the lycopene production might be increased. The lycopene yield of YSy221 and YSy222, which both have ~4 copies of the pathway, is of 13.14 mg/L and 14.55 mg/L, respectively (Figure 3B). For culture condition, the integration of lycopene in the membrane is known to reduce unsaturated fatty acids and cause stress. Interestingly, under low temperatures, yeast is known to increase membrane fluidity by increasing unsaturation of lipid acyl chains [53]. Thus, we tested impacts of lowering the fermentation temperature to the lycopene yields in the initial unSCRaMbLE, SCRaMbLEd strain, and screened strains with pathway integrated in the rDNA array. By lowering to 20 °C, all strains showed a further increase of the lycopene production, ranging from 41% to 185% (Figure 3B).

Comparing with the initial strain YSy200, the combined efforts of pathway and host strain optimization result in a maximal 129.5-fold improvement in the lycopene yield. All the SCRaMbLEd strains showed slightly higher OD at the stationary phase, but with no major increase in the doubling time compared with their parental unSCRaMbLE strain YSy200 (Figure S4). In general, the fitness in all our engineered strains is well-maintained. Our result suggests that combinatorial assembly of lycopene synthesis pathway can facilitate the improvement of stability and the genetic variations created by SCRaMbLE can enhance the compatibilities between the exogenous lycopene pathway and the host strain.

## 4. Discussion

Native cellular metabolism has been shaped by evolution, which is tightly regulated to benefit the survival. To achieve an industrial optimized strain, recurrent engineering is tedious and can be limited by the numbers of marker and marker recycling. In addition, it is time-consuming and requires prior knowledge to assist the targeted engineering. The highest lycopene yield up to date in *Saccharomyces cerevisiae* is achieved by extensive rational engineering efforts against multiple targeted genes. It also requires massive prior knowledge of metabolic networks of the host strain. In contrast, systematic approaches such as transcription machinery engineering, genome shuffling, and random mutagenesis can serve as alternative strategies to create genetic diversities and advance the strain performances [40]. Among them, SCRaMbLE served as a unique tool in the synthetic chromosome yeast. Here, we successfully demonstrated how SCRaMbLE creates genetic variations to host the exogenous lycopene pathway, and omics analysis allow us to identify combinatorial factors that contribute to the lycopene production. The most optimized strain YSy201 showed a 15.8-fold increasement with one round of SCRaMbLE, which only takes a few weeks of lab work. Our result shows that SCRaMbLE can accelerate the host strain optimization, which serve as a useful tool for the optimization of other toxic chemicals biosynthesis system.

The majority of metabolic pathways producing a target natural product require multiple genes to function coordinately. The rapid development of DNA de novo synthesis and assembly methods have provided the community with great tool kits to quickly build large DNA constructs. In addition, due to the fast advancing of DNA sequencing, more and more functional biological parts have been characterized and further standardized for the development of modular designed biological systems in various applications [30,54,55,56]. In our study, we took the advantages of both well-characterized biological parts and the well-established method for combinatorial assembly and successfully screened out constructs with increased lycopene production. Moreover, most importantly, the stability of the pathway in the host strain is properly maintained, which is even more important for industrial application. It is also true that the construct we screened out might not represent the optimal expression ratio of *IDI*, *CrtI*, *CrtB*, and *CrtE* to achieve the maximum yield due to the limited number of regulatory elements we used and the assembly efficiency of biological parts. However, our result demonstrated the potential of this strategy and could be further developed for metabolic engineering applications. In addition to genetic alterations that affect the yield of desirable metabolites, fermentation processes take important parts in. In our study, lowering the cultivation temperature will increase the unsaturation of lipid acyl chain in the cell membrane [53]. The increasement of unsaturation index can improve membrane fluidity and adaptability to environmental stresses [53]. We took this temperature induced membrane modification and explored how it could help to increase the lycopene yield. Through this approach, we further improved the lycopene yield by average 118% regardless the genetic backgrounds.

To sum up, in this study we provided a systematic approach for improving biosynthesis of target natural product through optimizing the yield and stability of the biosynthesis pathway by combinatorial assembly and the compatibility of the host strain by SCRaMbLE. We successfully demonstrated the feasibility of this high-effective strategy on the case study of lycopene biosynthesis, for which the final yield is 41.47 mg/L, increased by 129.5-fold in comparison with the initial strain, in which the lycopene yield is only 0.32 mg/L. We also noticed that the lycopene pathway in the SCRaMbLEd strain is relatively more stable than in the initial strains, which suggested that the genetic variation created by SCRaMbLE can enhance the compatibilities between the exogenous lycopene pathway and the host strain (Figure S5). With the increase of new knowledge generated from the trans-omics level discovery, and the fast advancing of genome writing and engineering technologies, our study provides researchers in the bioengineering field with a strategy to optimize a new, heterologous pathway biosynthesis system, by taking both pathway and host strain optimization into account, in a quick and cost-effective manner.

## Data Availability

The WGS and RNAseq data presented in this study are openly available in CNSA (China National GeneBank Nucleotide Sequence Archive, https://db.cngb.org/cnsa/) under project numbers CNP0001391.

## Supplementary Materials

The following are available online at https://www.mdpi.com/2306-5354/8/1/14/s1, all of the supplementary figures and tables are as follows: Figure S1. Overview of mevalonate pathway (MVA) biosynthesis mevalonate pathway in *S. cerevisiae*; Figure S2. Evaluation of cell fitness in unSCRaMbLE strain YSy200 and in SCRaMbLE strain YSy201; Figure S3. Colony PCR elucidated the instability of *CrtI* gene, which leads to the loss of lycopene; Figure S4. Evaluation of cell fitness in SCRaMbLEd strains (YSy201) and its derived strains (YSy221 and YSy222) with the initial host strain (YSy 200); Figure S5. Evaluation of pathway stability in SCRaMbLEd strains (YSy201) and its derived strains (YSy221 and YSy222) with the initial host strain (YSy200) by proportion of red colonies (%); Supplementary Table S1. The yeast strains used in this study; Supplementary Table S2. The plasmids used in this study and Supplementary Table S3. The primers used in this study; Supplementary Table S4. Detailed structure variations (SV) observed in synthetic chromosome II of YSy201 strain; Supplementary Table S5. RNA-Seq analysis of differentially expressed genes in YSy201 strain.
