# Supplementary material for: Systematical Engineering of Synthetic Yeast for Enhanced Production of Lycopene
Source: Bioengineering (Basel). 2021 Jan 19;8(1):14. doi: 10.3390/bioengineering8010014 (PMC7833358; doi:10.3390/bioengineering8010014)
Supplement: Supplementary file 1 [file bioengineering-08-00014-s001.pdf]

Supplementary materials

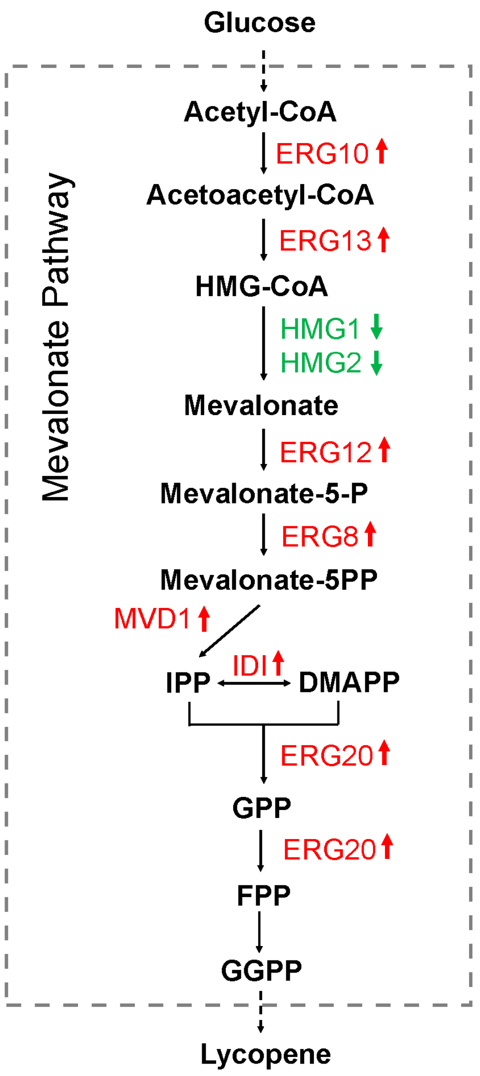

Figure S1. Overview of mevalonate pathway (MVA) biosynthesis mevalonate pathway in *S. cerevisiae*. The up-regulated genes are labeled in red and the down-regulated genes are labeled in green. .

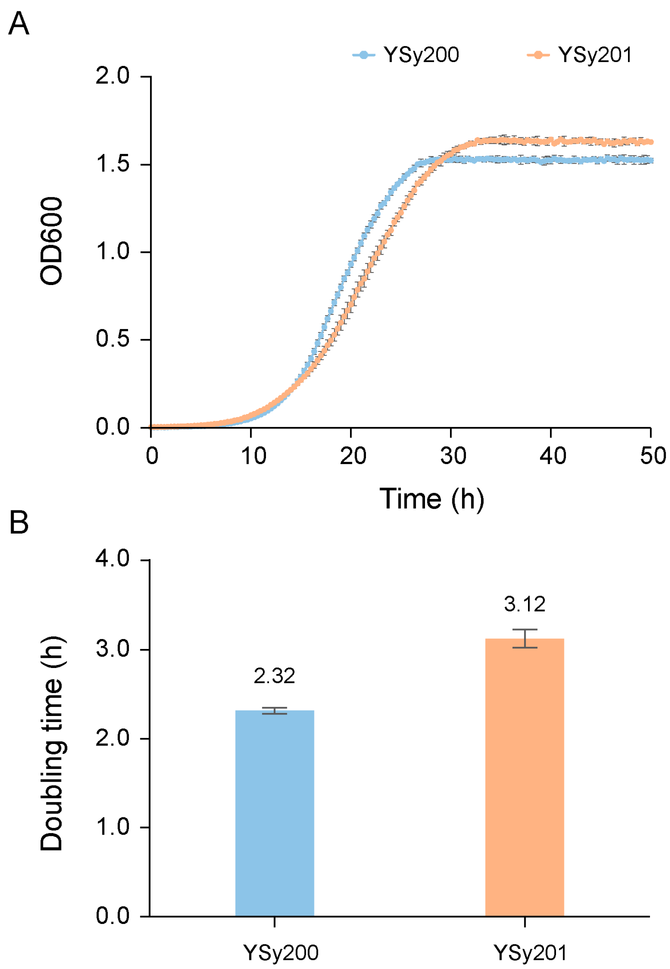

Figure S2. Evaluation of cell fitness in unSCRaMbLE strain YSy200 and in SCRaMbLE strain YSy201. A. Growth curves of YSy200 and YSy201 strain. B. Doubling time of YSy200 and YSy201. Three biological replicates were measured. The mean of three biological replicates of each strain was shown. Error bar represents the standard deviation.

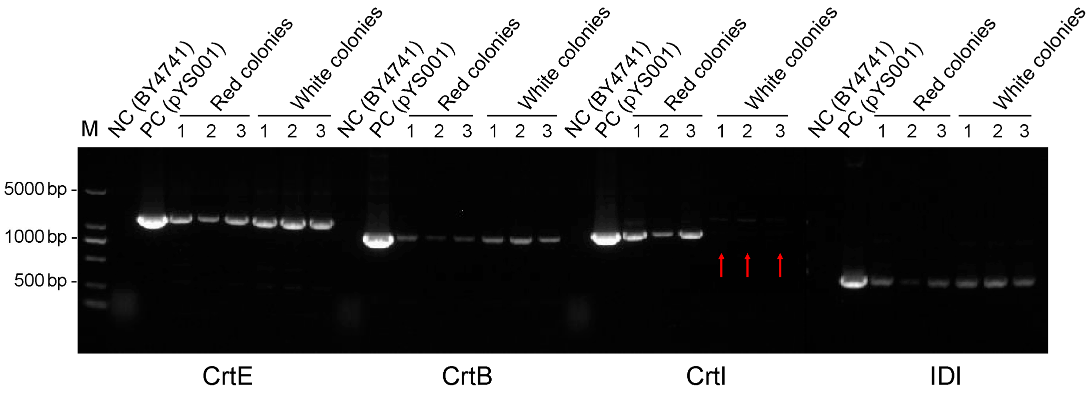

Figure S3. Colony PCR elucidated the instability of *CrtI* gene which leads to the loss of lycopene. M: DNA marker. NC: BY4741 used as negative control. PC: pYS001 used as positive control. Three biological repeats are randomly chosen from red and white colonies.

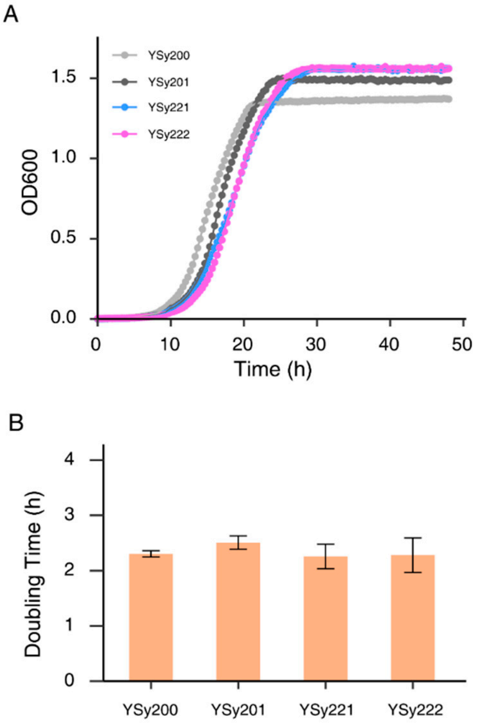

Figure S4. Evaluation of cell fitness in SCRaMbLEd strains (YSy201) and its derived strains (YSy221 and YSy222) with the initial host strain (YSy200). A. Growth curves of strains described above. B. Doubling time of strains described above. Error bars indicate ± SD (n = 3).

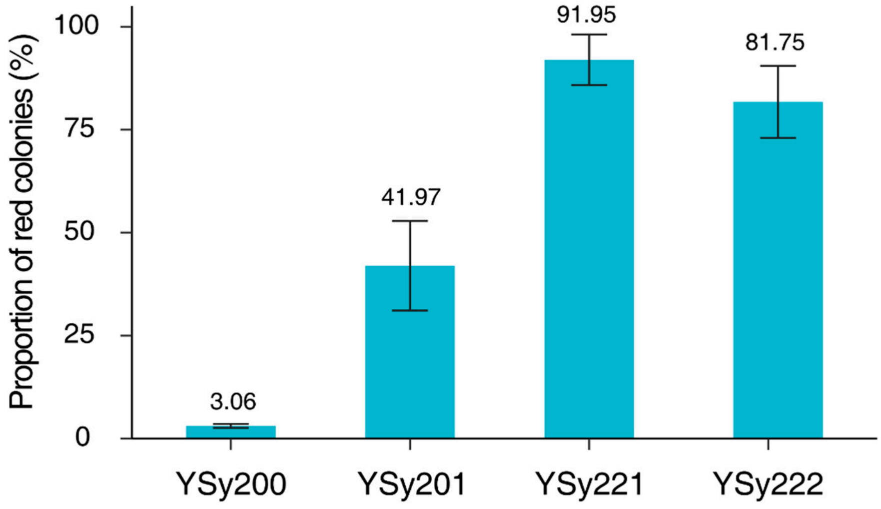

Figure S5. Evaluation of pathway stability in SCRaMbLEd strains (YSy201) and its derived strains (YSy221 and YSy222) with the initial host strain (YSy200) by proportion of red colonies (%). Error bars indicate ± SD (n = 3).

Table S1. The yeast strains used in this study.

Strains ID	Genotype	Description
BY4741	*MAT***a** *his3Δ1 leu2Δ0 LYS2 met15Δ0 ura3Δ0*	
BY4742	*MATα his3Δ1 leu2Δ0 lys2Δ0 MET15 ura3Δ0*	
synII	*MAT***a** *his3Δ1 leu2Δ0 LYS2 met15Δ0 ura3Δ0*	Strain constructed in previous study[16]
Diploid	*MAT***a***/α his3Δ1/his3Δ1 leu2Δ0/leu2Δ0 LYS2/lys2Δ0 met15Δ0/MET15 ura3Δ0/ura3Δ0*	synII mating to BY4742
YSy200	*MAT***a***/α his3Δ1/his3Δ1 leu2Δ0/leu2Δ0 LYS2/lys2Δ0 met15Δ0/MET15 ura3Δ0/ura3Δ0* pYS001	Diploid/ pYS001
YSy201	*MAT***a***/α his3Δ1/his3Δ1 leu2Δ0/leu2Δ0 LYS2/lys2Δ0 met15Δ0/MET15 ura3Δ0/ura3Δ0* pYS001	SCRaMbLEd strain from YSy200
YSy202	*MAT***a***/α his3Δ1/his3Δ1 leu2Δ0/leu2Δ0 LYS2/lys2Δ0 met15Δ0/MET15 ura3Δ0/ura3Δ0 pex32Δ0/PEX32*	YSy200/ *pex32Δ0/PEX32*
YSy203	*MAT***a** *his3Δ1 leu2Δ0 LYS2 met15Δ0 ura3Δ0* pYS001	BY4741/pYS001
YSy204	*MAT***a** *his3Δ1 leu2Δ0 LYS2 met15Δ0 ura3Δ0* pYS002	BY4741/pYS002
YSy205	*MAT***a** *his3Δ1 leu2Δ0 LYS2 met15Δ0 ura3Δ0* pYS003	BY4741/pYS003
YSy206	*MAT***a** *his3Δ1 leu2Δ0 LYS2 met15Δ0 ura3Δ0* pYS004	BY4741/pYS004
YSy207	*MAT***a** *his3Δ1 leu2Δ0 LYS2 met15Δ0 ura3Δ0* pYS005	BY4741/pYS005
YSy208	*MAT***a** *his3Δ1 leu2Δ0 LYS2 met15Δ0 ura3Δ0* pYS006	BY4741/pYS006
YSy209	*MAT***a** *his3Δ1 leu2Δ0 LYS2 met15Δ0 ura3Δ0* pYS007	BY4741/pYS007
YSy210	*MAT***a** *his3Δ1 leu2Δ0 LYS2 met15Δ0 ura3Δ0* pYS008	BY4741/pYS008
YSy211	*MAT***a** *his3Δ1 leu2Δ0 LYS2 met15Δ0 ura3Δ0* pYS009	BY4741/pYS009
YSy212	*MAT***a** *his3Δ1 leu2Δ0 LYS2 met15Δ0 ura3Δ0* pYS010	BY4741/pYS010
YSy213	*MAT***a** *his3Δ1 leu2Δ0 LYS2 met15Δ0 ura3Δ0* pYS011	BY4741/pYS011
YSy214	*MAT***a** *his3Δ1 leu2Δ0 LYS2 met15Δ0 ura3Δ0* pYS012	BY4741/pYS012
YSy221	*MAT***a***/α his3Δ1/his3Δ1 leu2Δ0/leu2Δ0 LYS2/lys2Δ0 met15Δ0/MET15 ura3Δ0/ura3Δ0 rDNA::KanMX-CCW12p-CrtE-ADH1t-CCW12p-CrtB-ADH1t-CCW12-CrtI-ADH1t-TDH3p-IDI-ADH1t*	YSy201 rDNA integration
YSy222	*MAT***a***/α his3Δ1/his3Δ1 leu2Δ0/leu2Δ0 LYS2/lys2Δ0 met15Δ0/MET15 ura3Δ0/ura3Δ0 rDNA::KanMX-CCW12p-CrtE-ADH1t-CCW12p-CrtB-ADH1t-CCW12-CrtI-ADH1t-TDH3p-IDI-ADH1t*	

Table S2. The plasmids used in this study.

Plasmids	Description	Source
HcKan_O	ORF receiving vector	
HcKan_P	promoter receiving vector	
HcKan_T	terminator receiving vector	Constructed in previous study [17]
POT	transcription unit receiving vector	
pRS416	metabolic pathway receiving vector	
CrtE-HcKan_O	HcKan_O carrying codon-optimized *CrtE* from *X.dendrorhous*	
CrtB-HcKan_O	HcKan_O carrying codon-optimized *CrtB* from *P.ananatis*	
CrtI-HcKan_O	HcKan_O carrying codon-optimized *CrtI* from *X.dendrorhous*	
IDI-HcKan_O	HcKan_O carrying codon-optimized *IDI* from *E. coli*	
TEF2p-HcKan_P	HcKan_P carrying *TEF2p* promoter	
ADH1t-HcKan_T	HcKan_T carrying *ADH1t* terminator	Constructed in this study
CrtE-POT	POT carrying *TEF2p-CrtE-ADH1t*	
CrtB-POT	POT carrying *TEF2p-CrtB-ADH1t*	
CrtI -POT	POT carrying *TEF2p-CrtI-ADH1t*	
IDI-POT	POT carrying *TEF2p-IDI-ADH1t*	
pYS001	pRS416 carrying *TEF2p-CrtE-ADH1t-TEF2p-CrtI-ADH1t-TEF2p-CrtB-ADH1t-TEF2p-IDI-ADH1t*	
pRS413-Cre/EBD	pRS413 carrying Cre recombinase expression cassette fused with an estradiol-binding domain (EBD)	Constructed in previous study [10]
CYC1p-HcKan_P	HcKan_P carrying *CYC1p* promoter	
TDH3p-HcKan_P	HcKan_P carrying *TDH3p* promoter	
CCW12p-HcKan_P	HcKan_P carrying *CCW12p* promoter	
PGK1p-HcKan_P	HcKan_P carrying *PGK1p* promoter	
RPL18bp-HcKan_P	HcKan_P carrying *RPL18bp* promoter	
CIT1t-HcKan_T	HcKan_T carrying *CIT1t* terminator	
FUM1t-HcKan_T	HcKan_T carrying *FUM1t* terminator	
ERP2t-HcKan_T	HcKan_T carrying *ERP2t* terminator	
pYS002	pRS416 carrying *CYC1p-CrtE-ADH1t-CYC1p-CrtB-ADH1t-CYC1p-CrtI-ADH1t-CYC1p-IDI-ADH1t*	
pYS003	pRS416 carrying *RPL18bp-CrtE-ADH1t-RPL18bp-CrtB-ADH1t-RPL18bp-CrtI-ADH1t-RPL18bp-IDI-ADH1t*	Constructed in this study
pYS004	pRS416 carrying *CCW12p-CrtE-ADH1t-CCW12p-CrtB-ADH1t-CCW12p-CrtI-ADH1t-CCW12p-IDI-ADH1t*	
pYS005	pRS416 carrying *CYC1p-CrtE-ADH1t-RPL18bp-CrtB-ADH1t-CYC1p-CrtI-ADH1t-CYC1p-IDI-ADH1t*	
pYS006	pRS416 carrying *CYC1p-CrtE-ADH1t-CCW12p-CrtB-ADH1t-CYC1p-CrtI-ADH1t-CYC1p-IDI-ADH1t*	
pYS007	pRS416 carrying *CYC1p-CrtE-ADH1t-CYC1p-CrtB-ADH1t-CYC1p-CrtI-ADH1t-RPL18bp-IDI-ADH1t*	
pYS008	pRS416 carrying *CYC1p-CrtE-ADH1t-CYC1p-CrtB-ADH1t-CYC1p-CrtI-ADH1t-PGK1p-IDI-ADH1t*	
pYS009	pRS416 carrying *PGK1p-CrtE-ADH1t-PGK1p-CrtB-ADH1t-PGK1p-CrtI-ADH1t-TDH3p-IDI-ADH1t*	

pYS010	pRS416 carrying *CCW12p-CrtE-ADH1t-CCW12p-CrtB-ADH1t-CCW12p-CrtI-ADH1t-TDH3p-IDI-ADH1t*
pYS011	pRS416 carrying *CYC1p-CrtE-ERP2t-CYC1p-CrtB-CIT1t-TEF2p-CrtI-FUM1t-CCW12p-IDI-ADH1t*
pYS012	pRS416 carrying *CYC1p-CrtE-ERP2t-CCW12p-CrtB-CIT1t-TDH3p-CrtI-FUM1t-TDH3p-IDI-ADH1t*

Table S3. The primers used in this study.

**primers**	**Sequences (5′-3′)**
MAT locus 1	AGTCACATCAAGATCGTTTATGG
MATalpha locus 2	GCACGGAATATGGGACTACTTCG
MATa locus 2	ACTCCACTTCAAGTAAGAGTTTG
TEF2p-F	AGCGTGGGTCTCAGGCTGGGCGCCATAACCAAG
TEF2p -R	GTGCTGGGTCTCGCATCTTTAGTTAATTATAGTTCGTTGACCGTATATTC
ADH1t-F	AGCGTGGGTCTCATAGCCGAATTTCTTATGATTTATGAT
ADH1t-R	GTGCTGGGTCTCGGAGGCCGGTAGAGGTGTGG
HcKan-seq-F	GATCCTTTGATTTTCTACCG
HcKan-seq-R	CTCGATAACTCAAAAAATACG
POT-seq-F	CTTTCCTGCGTTATCCCCTGATTC
POT-seq-R	GATGTGCTGCAAGGCGATTAAG
pRS416-seq-F	CGGAGCCTATGGAAAAACGCC
pRS416-seq-R	TCTTCCGCTTCCTCGCTCAC
Cre-seq-F	GTCCAATTTACTGACCGTAC
Cre-seq-R	GGCTATACGTAACAGGGTG
CCW12p-F	AGCGTGGGTCTCAGGCTCTATTGGCGTCTGATTTCCG
CCW12p-R	GTGCTGGGTCTCGCATCTTATTGATATAGTGTTTAAGCGAATGAC
PGK1p-F	AGCGTGGGTCTCAGGCTGTTTGCAAAAAGAACAAAACTG
PGK1p-R	GTGCTGGGTCTCGCATCCATTGTTTTATATTTGTTGTAAAAAGT
RPL18bp-F	AGCGTGGGTCTCAGGCTTTAGAACGCTCCTGTAAC
RPL18bp-R	GTGCTGGGTCTCGCATCTTTTGTTTTTTGTTTTCTTCTAATTG
TDH3p-F	AGCGTGGGTCTCAGGCTTCATTATCAATACTGCCATTTC
TDH3p-R	GTGCTGGGTCTCGCATCTTTGTTTGTTTATGTGTGTTTATTCG
CYC1p-F	AGCGTGGGTCTCAGGCTTTCATTTGGCGAGCGTTGG
CYC1p-R	GTGCTGGGTCTCGCATCTTAGTGTGTGTATTTGTGTTTGTGTGTC
rDNA25-HoL-F	ACAAATCAGACAACAAAGGCT
rDNA25-HoL-KANMX-R	ATTCTGGGCCTCCATGTCAGCAGAATTCGGTAAGCG
KANMX-F	CGCTTACCGAATTCTGCTGACATGGAGGCCCAGAAT
KANMX-CCW12p-R	CAAAACGGAAATCAGACGCCAATAGAGCCAGGTAGAGCAGTATAGCGACCAGCATTCAC
ADH1t-rDNA25-HoR-F	TCTTATTGACCACACCTCTACCGGCCTCTGAGACCTGCTGGCCCAGTGAAATGC
rDNA25-HoR-R	GTTTGACCTCAAATCAGGTAGG
CrtE-seq-F	GCTTTCAACTACTGGTTGGACG
CrtE-seq-R	CAATGGGATGTCAGCCAACT
CrtB-seq-F	ATGAATAATCCGTCGTTACTCAATC
CrtB-seq-R	CTACTAGAGCGGGCGCT
CrtI-seq-F	GCTAAGTTCAACTTCAACGCTC
CrtI-seq-R	GAAAGCCAAAACACCAACAGATC

IDI-seq-F	ATGCAAACGGAACACGTCAT
IDI-seq-R	TAATTGTGCTGCGCGAAAGC
CrtE-qPCR-F	CCCACAAACCATCAACACCG
CrtE-qPCR-R	AACTGGCATTGGGATTGGGG
CrtB-qPCR-F	TTACAAACGCCCGAACAACG
CrtB-qPCR-R	TTCCTGAAAAGCCGCAAACG
CrtI-qPCR-F	ACGCTGACTTGGTTTACGCT
CrtI-qPCR-R	AAGTCAGCCCACCAAGATCG
IDI-qPCR-F	CCTGGCGTGTGGACTAACTC
IDI-qPCR-R	CAAGCTCATAACGGCAACGG
ACT1-qPCR-F	ATGGATTCTGAGGTTGCTGCT
ACT1-qPCR-R	TGGTGTCTTGGTCTACCGAC
RFA1-qPCR-F	GTGTGACGGATTTTGGTGGC
RFA1-qPCR-R	TGATGAAGTTTGCGTTGCGG

Table S4. Detailed structure variations (SV) observed in synthetic chromosome II of YSy201 strain. DUP for duplication, DEL for deletion, INV for inversion.

SV_Type	Coordination Start	Coordination End
DUP	1	19985
DEL	19952	63587
DUP	63554	118366
DEL	118333	212941
INV	270502	271873
INV	317884	320723
DEL	460960	469144
DEL	540417	573406
DEL	581558	581823
DEL	607862	608082
DEL	684169	770035

Table S5. RNA-Seq analysis of differentially expressed genes in YSy201 strain.

Systematic Name	Standard Name	log2FoldChange	Up/Down-Regulation
YAL004W	-	1.22	up
YAR009C	-	-1.58	down
YAR010C	-	-1.53	down
YBL005W-A	-	-1.54	down
YBL005W-B	-	-1.32	down
YBL009W	*ALK2*	1.52	up
YBL043W	*ECM13*	-2.97	down
YBL044W	-	-1.91	down
YBL053W	-	1.28	up

YBL058W	*SHP1*	1.20	up
YBL059C-A	*CMC2*	1.12	up
YBL062W	-	1.98	up
YBL063W	*KIP1*	1.14	up
YBL068W-A	-	4.58	up
YBL072C	*RPS8A*	1.07	up
YBL077W	-	2.69	up
YBL083C	-	1.51	up
YBL087C	*RPL23A*	1.01	up
YBL092W	*RPL32*	1.12	up
YBL107C	-	2.18	up
YBR012W-B	-	-1.14	down
YBR054W	*YRO2*	-1.52	down
YBR127C	*VMA2*	-1.00	down
YBR145W	*ADH5*	-1.05	down
YBR168W	*PEX32*	-1.05	down
YBR169C	*SSE2*	-1.79	down
YBR176W	*ECM31*	-1.16	down
YBR180W	*DTR1*	-1.03	down
YBR182C	*SMP1*	-1.21	down
YBR182C-A	-	-1.13	down
YBR183W	*YPC1*	-1.77	down
YBR249C	*ARO4*	-1.54	down
YBR250W	*SPO23*	-1.62	down
YBR255C-A	-	-1.24	down
YBR255W	*MTC4*	-1.08	down
YBR269C	*FMP21*	-1.03	down
YBR270C	*BIT2*	-1.27	down
YBR276C	*PPS1*	-1.05	down
YBR285W	-	-1.58	down
YBR286W	*APE3*	-1.14	down
YBR289W	*SNF5*	-1.13	down
YBR293W	*VBA2*	-1.25	down
YBR294W	*SUL1*	-1.67	down
YCL019W	-	-1.26	down
YCL020W	-	-1.93	down
YCL025C	*AGP1*	-1.27	down
YCR018C	*SRD1*	-1.05	down
YDL021W	*GPM2*	-1.28	down
YDL086C-A	-	1.23	up
YDL151C	*BUD30*	1.16	up
YDL246C	*SOR2*	1.07	up
YDR015C	-	-1.43	down
YDR034C-D	-	-1.05	down
YDR098C-B	-	-1.06	down
YDR119W-A	-	-1.25	down
YDR210C-D	-	-1.13	down
YDR210W	-	1.01	up

YDR261C-C	-	-1.25	down
YDR261C-D	-	-1.24	down
YDR277C	*MTH1*	-1.26	down
YDR342C	*HXT7*	-1.46	down
YDR343C	*HXT6*	-1.13	down
YDR365W-A	-	-1.49	down
YDR365W-B	-	-1.33	down
YDR544C	-	1.11	up
YIL057C	*RGI2*	-1.36	down
YIL177C	-	-1.07	down
Q0070	*AI5_ALPHA*	-5.51	down
YEL011W	*GLC3*	-1.02	down
YEL065W	*SIT1*	-1.71	down
YER053C-A	-	-1.28	down
YER062C	*HOR2*	-1.01	down
YER066C-A	-	-1.04	down
YER137C-A	-	-1.67	down
YER138C	-	-1.69	down
YER138W-A	-	-1.08	down
YER142C	*MAG1*	1.19	up
YER150W	*SPI1*	-1.14	down
YER160C	-	-1.40	down
YER175C	*TMT1*	-1.18	down
YFL002W-A	-	-1.36	down
YFL011W	*HXT10*	1.36	up
YFL054C	-	-1.12	down
YFR015C	*GSY1*	-1.10	down
YFR017C	-	-1.21	down
YFR053C	*HXK1*	-1.61	down
YFR057W	-	1.49	up
YGL117W	-	-1.01	down
YGL123C-A	-	-5.20	down
YGL158W	*RCK1*	-1.03	down
YGL256W	*ADH4*	-2.20	down
YGL258W	*VEL1*	-1.32	down
YGR022C	-	-1.07	down
YGR027W-B	-	-1.13	down
YGR038C-B	-	-1.19	down
YGR109C	*CLB6*	1.23	up
YGR161C-D	-	-1.21	down
YGR161W-B	-	-1.12	down
YGR164W	-	1.07	up
YGR243W	*FMP43*	-1.34	down
YHL030W	*ECM29*	1.01	up
YHR092C	*HXT4*	-1.57	down
YHR139C	*SPS100*	1.25	up
YHR214C-B	-	-1.75	down
YJL052W	*TDH1*	-1.75	down

YJL116C	*NCA3*	-1.40	down
YJL130C	*URA2*	-1.02	down
YJL133C-A	-	-1.32	down
YJL225C	-	-1.04	down
YJR005C-A	-	1.37	up
YJR009C	*TDH2*	-1.17	down
YJR027W	-	-1.35	down
YJR028W	-	-1.83	down
YJR029W	-	-1.26	down
YJR038C	-	-1.14	down
YKL001C	*MET14*	1.11	up
YKL083W	-	1.28	up
YKL156C-A	-	1.86	up
YKL163W	*PIR3*	-1.01	down
YKR034W	*DAL80*	-1.62	down
YKR035W-A	*DID2*	1.57	up
YKR039W	*GAP1*	-1.18	down
YKR075C	-	-1.61	down
YLR035C-A	-	-1.39	down
YLR136C	*TIS11*	-1.12	down
YLR157C-B	-	-1.48	down
YLR227W-B	-	-1.52	down
YLR256W-A	-	-1.63	down
YLR303W	*MET17*	1.10	up
YLR367W	*RPS22B*	-1.09	down
YLR410W-A	-	-1.29	down
YLR437C-A	-	-1.15	down
YLR438W	*CAR2*	-1.37	down
YLR466C-B	-	1.12	up
YML039W	-	-1.51	down
YML040W	-	-1.34	down
YML045W	-	-1.29	down
YML045W-A	-	-1.13	down
YMR011W	*HXT2*	-1.02	down
YMR045C	-	-1.62	down
YMR046C	-	3.39	up
YMR050C	-	-1.44	down
YMR058W	*FET3*	-1.20	down
YMR081C	*ISF1*	-1.86	down
YMR105C	*PGM2*	-1.26	down
YMR194C-B	*CMC4*	-1.11	down
YMR206W	-	-1.11	down
YNL040W	-	-1.02	down
YNL144C	-	-1.10	down
YNL150W	-	1.08	up
YNL155W	-	1.21	up
YNL160W	*YGP1*	-1.23	down
YNL284C-B	-	-1.37	down

YNL339W-A	-	-2.20	down
YNR014W	-	-1.21	down
YNR056C	*BIO5*	-1.46	down
YOL014W	-	1.42	up
YOL052C-A	*DDR2*	-1.61	down
YOL103W-B	-	-1.30	down
YOL104C	*NDJ1*	-1.23	down
YOL154W	*ZPS1*	-2.18	down
YOL162W	-	1.15	up
YOR108C-A	-	1.22	up
YOR142W-A	-	-2.49	down
YOR142W-B	-	-1.38	down
YOR178C	*GAC1*	-1.24	down
YOR192C-B	-	-1.04	down
YOR277C	-	3.90	up
YOR382W	*FIT2*	-1.32	down
YPL081W	*RPS9A*	-1.11	down
YPL111W	*CAR1*	-1.15	down
YPL257W-A	-	-1.45	down
YPL257W-B	-	-1.10	down
YPL265W	*DIP5*	-1.47	down
YPR137C-A	-	-1.10	down
YPR137C-B	-	-1.15	down
YPR138C	*MEP3*	-1.04	down
YPR145C-A	-	1.22	up
YPR158C-D	-	-1.46	down
YPR158W-B	-	-1.62	down
YPR160W	*GPH1*	-1.02	down
